# Does China’s competitive generic substitution policy deliver equivalent clinical outcomes? A pilot study with two generic formulations of olanzepine

**DOI:** 10.3389/fphar.2023.1097600

**Published:** 2023-02-22

**Authors:** Chao Zhang, Yudan Ding, Zhenzhen Wu, Juan Wang, Xiangping Wu, Weiwei Xie

**Affiliations:** ^1^ Ningbo Kangning Hospital, Ningbo, Zhejiang, China; ^2^ Department of Psychiatry, National Clinical Research Center for Mental Disorders, The Second Xiangya Hospital of Central South University, Changsha, Hunan, China

**Keywords:** generic olanzapine, volumed-based drug procurement policy, serum concentration, clinical effect, safety

## Abstract

With the National Centralized Drug Procurement policy gradually applied nationally in China, concerns about the effectiveness and safety of bid-winning generic drugs are growing again, but relevant studies are lacking. This real-world, before-and-after study was conducted to explore the clinical effects of switching between two versions of generic olanzapine (one of them was bid-winning product). Pre-and post-switching serum olanzapine concentrations were compared. A total of 30 patients were included and results showed the log-transformed, dose-adjusted concentration of bid-winning generic olanzapine was significantly lower than that of another generic olanzapine, while no significant differences were shown on Clinical Global Impressions Severity of Illness or Improvement ratings before and after switching. This study suggest that a generic version of a psychotropic medication may not be of therapeutic equivalence or bioequivalence with another generic one. Changes in efficacy or tolerability are possible in every switch. Therapeutic drug monitoring could be a valuable tool during switches between generic drugs. Larger prospective clinical studies for other generic psychotropic medications in target populations are warranted.

## 1 Introduction

The ever-increasing use of medicines and development of new innovative medicines have increased the life expectancy of populations and the health expenditure worldwide, placing great pressure on healthcare budgets of many countries and personal finances of patients. In China, the annual growth in real health spending (the sum of expenditure on all the core healthcare functions, including total healthcare services, medical goods dispensed to outpatient, prevention and public health services, and health administration and health insurance) has been much faster than the growth in its gross domestic product, with 11.5% *versus* 8.5% in 2009–2015 (OECD and World Health Organization, 2012) In particular, pharmaceutical expenditure makes up a large share of the total cost at 32.39% in 2018, much higher than the average level of any other country in the Organization for Economic Cooperation and Development (Zhai et al., 2020).

A key solution to mitigate drug expenditure is generic drug substitution. Once patent protection for the branded product has expired, generic alternatives that contain the same active ingredients enter the market with much lower prices [10%–80% lower than their brand name counterparts (Simoens and De Coster, 2006)]. Subsequently, fierce competition often leads to substantially lower prices for the original drug and its generic versions. Moreover, government procurement is a common activity to promote the use of generic drugs. The National Centralized Drug Procurement (NCDP), a volumed-based drug procurement policy, was implemented in Mainland China in 2019. The first round of this policy was piloted in 11 selected cities, and then spread nationally. Under this policy, an agreed annual procurement volume of each medicine (estimated with reference to the prescription volume in the previous year) in the procurement list should be submitted by public medical institutions. The government then organizes competitive bidding and price negotiation in accordance with the submitted procurement volume. Any public medical institution that does not complete the yearly agreed volume faces penalties, which, in most cases, are carried out by related department and individual doctor. Undoubtedly, the market structure of policy-related drugs inevitably is reshaped. The bid-winning drugs gradually dominate the market, and changes are seen in the market behavior of other pharmaceutical enterprises of policy-involved drugs. The Fisher Price Index (a common consumer price index measuring the drug price level over a given periods) of winning products remarkably reduced by 79.02% in the first-round pilot in Shenzhen (Wang et al., 2021). With the expanded scope of involved drugs, price reduction is expected to be observed to a greater extent and in a larger scope.

However, some physicians and patients express concerns about these bid-winning generic drugs. Although the increased availability and decreased cost are desirable, which are particularly important in patients with psychiatric illnesses due to the early onset and chronic illness duration, whether these drugs are always as safe and effective as brand-name drugs or whether switching from a generic product to another one is risky are unclear. After all, bioequivalence and pharmacological equivalence do not mean therapeutic equivalence that needs preclinical and clinical trials to establish (Borgheini, 2003; Seoane-Vazquez et al., 2016). Moreover, given that the bioequivalence criteria allow pharmacokinetic measures (i.e., the maximum plasma concentration and area under the curve of the serum concentration time curve) to vary from −20% to 25% between generic and reference drugs (Li et al., 2021), the potential variability between different generic products of a given original drug could be much greater, which may lead to loss of efficacy or reduced tolerability (Blier et al., 2019).

Several studies in different countries and areas have examined the clinical effect of brand-generic switches of antipsychotics. Most of them were retrospective studies, only a minority were randomized controlled studies, and the results were mixed. For instance, a retrospective study in New Zealand demonstrated that brand-generic switch could be safely conducted, and a high proportion of patients had multiple switching between generic products (Lessing et al., 2015). Another study from Italy including 25 patients reported a significant reduction in olanzapine concentrations after brand-generic switch, but no clinical deterioration was observed in 4 weeks (Italiano et al., 2015). Different generic versions of olanzapine may contribute to the inconsistency. To date, only one retrospective database study in Mainland China has reported the efficacy and safety of a generic olanzapine, which did not significantly differ from reference olanzapine (Yue et al., 2022). With the NCDP policy gradually applied nationally and an increasing number of patients being involved, assessing the effectiveness and safety of these bid-winning generic drugs in clinical samples and the clinical effects of brand-generic or generic-generic switch is imperative. This real-world study was conducted to investigate whether the switch from a generic olanzapine to another one in patients with psychiatric disorders could affect the therapeutic response, adverse events, and serum olanzapine concentrations.

## 2 Method

All patients aged older than 18 years, already stabilized on treatment (at least 6 months) and agreed to switch to another generic olanzapine, were recruited from NingBo Kangning Hospital, China (from December 2019 to April 2022). The exclusion criteria included severe or acute physical illness and recent changes in other drug treatments. All enrolled patients were chronically treated with a generic olanzapine (Olanzapine Tablets, HANSOH PHARMA, Jiangsu, China) and then switched to the same dose of the bid-winning generic olanzapine (Olanzapine, Dr. Reddy Laboratories Ltd., India). Serum olanzapine concentrations were evaluated before and after drug replacement when the steady-state conditions of olanzapine had been established (at least 4 weeks after switching to the second generic olanzapine). Each venous blood sample was obtained after an overnight fast. The Clinical Global Impressions (CGI) Scale was used to quantify and track the treatment response and symptom fluctuation over time. Self-reported side effects and abnormal laboratory assessments were recorded. Each subject was followed up for at least 3 months. The protocol received full approval from the local Ethics Committee, and a written informed consent was provided by each individual.

The serum concentrations of the two generic versions of olanzapine were analyzed *via* high-performance liquid chromatography in combination with mass spectroscopy (LC-MS), as described by Kirchherr and Kuhn-Velten (2006). The key advantages of this method are sensitivity and selectivity, together with time-saving sample preparation (Hiemke et al., 2011). The limit of assay quantification was 5 ng/mL. The LC-MS system used consisted of Prominence LC-20A (Shimadzu Corporation, Kyoto, Japan) and a mass spectrometry product ANAX FLC 2701 (ANAX, Hunan, China).

As the distributions of the olanzapine concentrations and dose-adjusted concentrations were heavily left-skewed, the log-transformed concentrations were used in the subsequent paired *t*-test. Fisher’s exact test was used to determine if the proportions of adverse events during treatment with the two generic versions significantly differ from each other. All statistical tests were two-tailed, with a significance level set at 0.05.

## 3 Results

**FIGURE 1:**
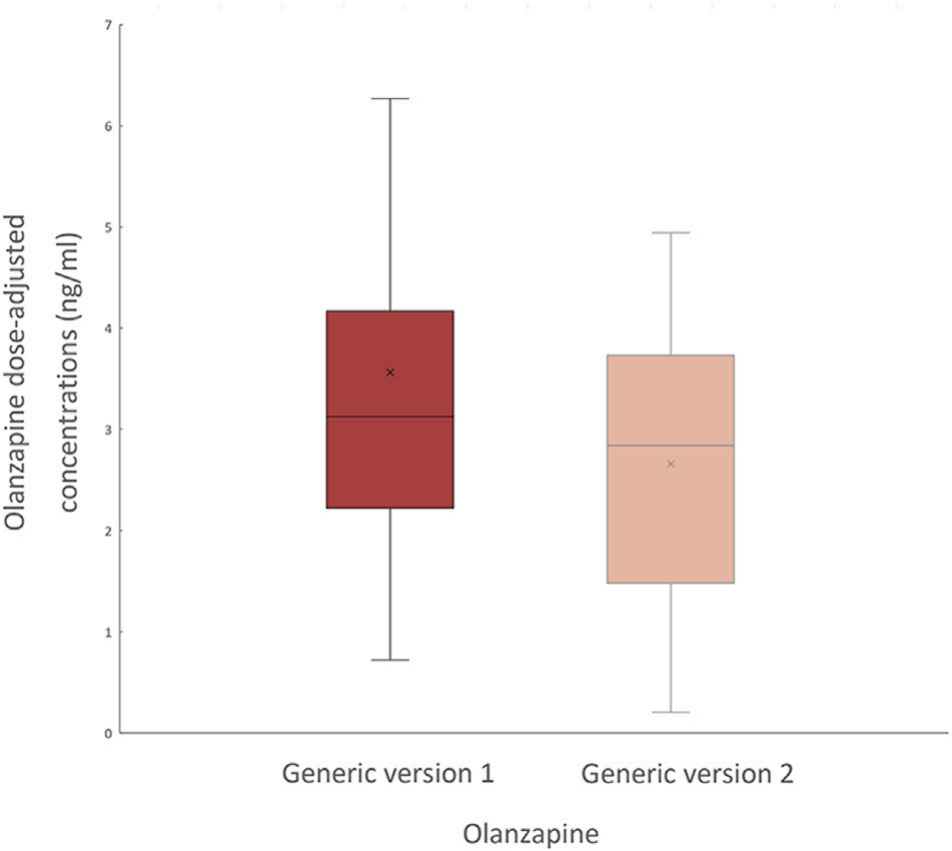
Steady-state olanzapine dose-adjusted concentrations before (generic version 1, the original generic olanzapine) and after (generic version 2, the bid-winning olanzapine) drug switch.

A total of 30 patients (16 men and 14 women; mean age of 49.8 ± 17.9 years) were recruited and completed follow-ups. Among them, 26 met the DSM-V or ICD-10 criteria for schizophrenia-spectrum disorders, one for bipolar disorder, one for major depressive disorder, and other two for neurocognitive disorders (Table 1). The mean olanzapine dose was 12.6 ± 5.5 mg/d. The median dose-adjusted concentrations of olanzapine before and after generic-generic switching were 3.12 and 2.84 ng/mL, respectively (Figure 1). The log-transformed, dose-adjusted concentration of bid-winning generic olanzapine was significantly lower than that of another generic olanzapine (*p* = 0.011).

**TABLE 1:** Demographic and clinical profiles of patients.

Variables			
Age/years		49.8 ± 17.9	
Gender (Male/Female)		16/14	
Diagnoses			
Schizophrenia-spectrum disorders		26	
Bipolar disorder		1	
Major depressive disorder		1	
Neurocognitive disorders		2	
CGI-S score	Baseline	3.2 ± 0.71	*p* = 0.103
	Follow up	3.1 ± 0.52	
CGI-I score	Baseline	2.8 ± 0.65	*p* = 0.573
	Follow up	2.9 ± 0.57	
Follow-up period/month (range)		3–15	
Dose of olanzapine/mg		12.6 ± 5.5	

Abbreviations: CGI-S, Clinical Global Impressions-Severity scale; CGI-I, Clinical Global Impressions-Improvement scale.

**FIGURE 2:**
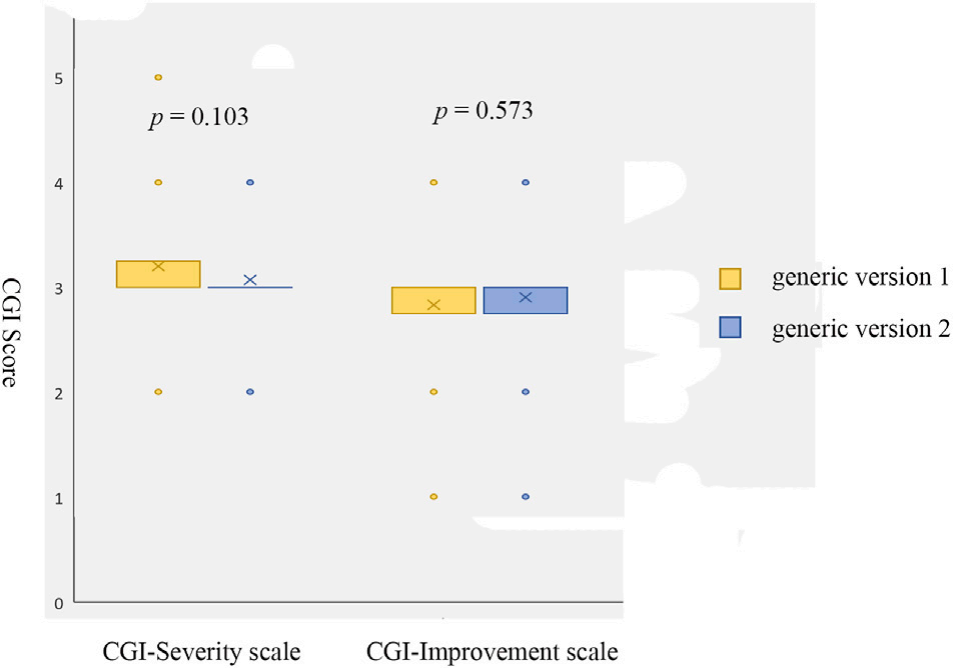
Scores of CGI Severity scale and Improvement scale before (generic version 1, the original generic olanzapine) and after (generic version 2, the bid-winning olanzapine) drug switch.

No significant differences were shown on CGI Severity of Illness (*p* = 0.103) or Improvement ratings (*p* = 0.573) before and after the replacement of bid-winning generic olanzapine (Figure 2). The proportions of adverse events reported during the treatment of original generic olanzapine were not different from that emerged after switching to the bid-winning one (Table 2).

**TABLE 2:** Adverse events reported before (generic version 1, the original generic olanzapine) and after (generic version 2, the bid-winning olanzapine) drug switch.

Adverse events	Generic version 1 (%)	Generic version 2 (%)	*p*
Self-reported side effects			
Constipation	6.67	6.67	—
Vomiting	0	0	—
Nausea	0	0	—
Headache	0	0	—
Dizziness	0	0	—
Insomnia	0	0	—
Rash	0	0	—
Extrapyramidal side effects	0	0	—
Laboratory assessments			
Decreased WBC/lymphocytes	0	3.45	1
Increased alanine aminotransferase/aspartate transferase	20	20	—
Elevated creatine kinase	30.43	50	0.567
Increased triglyceride	66.67	50	0.789
Increased total cholesterol	11.11	15.38	1
Increased low-density lipoprotein	12	21.74	0.705
Increased fasting glucose	11.11	20	0.706
Hyperprolactinemia	29.41	25	1
QT interval prolongation	0	0	—

## 4 Discussion

The results showed that the steady-state serum olanzapine concentrations were altered after conducting generic-generic switch in patients, whereas no clinical worsening of symptoms was observed during at least 3-month follow-up period. Similar to using the original generic olanzapine, some adverse events and mildly abnormal laboratory assessments were recorded after switching to the bid-winning generic olanzapine, but no patient withdrew or was required to be reverted to the former olanzapine due to side effects. This investigation underscored the potential effects of drug switches between the two generic versions of olanzapine, and together with previous studies, the results provide considerations for clinical management of brand-generic and generic-generic switches.

As mentioned above, although generic drugs generally must have the same active pharmaceutical ingredients as their reference products and demonstrate pharmacological equivalence and bioequivalence, they may have relatively different excipients and other alterations that could cause critical changes in drug stability, absorption, and tolerability (Borgheini, 2003). In addition, the current bioequivalence studies are generally performed in small groups of healthy volunteers with typically single-dose administration, which offer little reference to clinical practice. A notable detail that although up to 95% of all medicinal products in China are generic drugs, no consistency standard was established between generic products and their brand-name counterparts until 2016. Many generic drugs in the market at present are still in the process of consistency evaluation. Accordingly, larger clinical studies (especially prospective ones) in target populations are warranted to determine whether the public and governments could benefit from the NCDP policy, or conversely, more medical expenditures are expected due to relapse of symptoms and increased hospitalization rates.

When initiating or switching to generic psychotropic drugs, at first, an awareness that many factors, such as psychological, physiological, pharmacological, and interactional factors, could contribute to changes of in clinical status is needed, which may or may not be associated with the use of a certain generic product (Carbon and Correll, 2013). For example, published studies have indicated that alterations in characters of a medication (i.e., shape, color, and packaging) could lead to patients’ distrust and reduced adherence (Nuss et al., 2004; Liu et al., 2011). Negative concerns and perceptions regarding generic products in patients may also affect medication adherence (Straka et al., 2017). Thus, carefully communicating the treatment strategy of generic drugs with each patient is important. In addition, the findings from this study and those of previous studies showed that serum concentration measurement could be a valuable tool for titrating the dosage of targeted drugs, especially in brand-generic and generic-generic switches.

Several limitations of this study need to be mentioned. First of all, this is a preliminary study with only 30 patients recruited. The small sample size may be vulnerable for type 2 error and does not have adequate statistical power to detect any differences in treatment response and symptom fluctuation, and adverse events. Second, this study was conducted at a local hospital, and the results might not be generalizable to national patients with psychiatric disorders due to potentially different clinical practice and healthcare policies. Third, given that we did not include a control group, it is not necessarily possible to say whether the identified difference in this study might be due to chance. Fourth, some patients were followed up for just 3 months, it is possible that the length of time of follow-up for post-switching is shorter and has less of a chance of truly stabilizing compared to the length pre-switching period.

Until more clinical studies on Chinese people are available, the preliminary results suggest that a generic version of a psychotropic medication may not be of bioequivalence with another generic one (despite our preliminary results showed no clinical outcome changed after switching, given the small sample size, the conclusion that the two generics are of therapeutic equivalence should interpret with caution). Changes in efficacy or tolerability are possible in every switch. Thus, performing therapeutic drug monitoring is important. Furthermore, we strongly suggest that authorities should reveal pharmacological equivalence and bioequivalence data on all on-the-market generics and brand name drugs, and establish guidelines on subsequent assessments for therapeutic equivalence on clinical samples and procedures of surveillance for therapeutic inequivalence. More nation-wide and well-designed clinical studies for generic drugs are warranted.

## Data Availability

The raw data supporting the conclusion of this article will be made available by the authors, without undue reservation.
